# Comparison of *In Vitro* Antibacterial Activity of *Epaltes divaricata* and *Vetiveria zizanioides* against Methicillin-Resistant *Staphylococcus aureus*

**DOI:** 10.1155/2020/8239053

**Published:** 2020-07-14

**Authors:** Hasanga Rathnayake, Manikkuwadura Hasara Nethmini De Zoysa, Ruwani Punyakanthi Hewawasam, Weerasinghe Mudiyanselage Dilip Gaya Bandara Wijayaratne

**Affiliations:** ^1^Department of Biochemistry, Faculty of Medicine, University of Ruhuna, Matara, Sri Lanka; ^2^Department of Medical Laboratory Science, Faculty of Allied Health Sciences, University of Ruhuna, Matara, Sri Lanka; ^3^Department of Microbiology, Faculty of Medicine, University of Ruhuna, Matara, Sri Lanka

## Abstract

Methicillin-resistant *Staphylococcus aureus* (MRSA) is a major cause of hospital- and community-acquired infections worldwide. Therefore, this study was conducted to explore the antibacterial activity of the two medicinal plants *Epaltes divaricata* and *Vetiveria zizanioides* against strains of MRSA which were isolated from patients with skin and soft tissue infections. Hexane, ethanol, and water extracts of *E. divaricata* (whole plant) and *V. zizanioides* (roots) were prepared. Clinical isolates of MRSA strains (*n* = 20) were used for the study. Bacterial susceptibility was tested using a disc diffusion assay. Minimum inhibitory concentration (MIC) was determined by a broth microdilution method. Vancomycin was used as the positive control. Hexane, ethanol, and water extracts of *E. divaricata* showed inhibitory zones against MRSA. Except for water extract, both hexane and ethanol extracts of *V. zizanioides* showed inhibitory zones. MIC ranges of hexane, ethanol, and water extracts in *E. divaricata* were 0.012–0.32 mg/mL, 0.019–2.4 mg/mL, and 0.019–0.48 mg/mL, respectively. Respective MIC ranges of hexane and ethanol extracts of *V. zizanioides* were 0.003–0.032 mg/mL and 0.019–2.4 mg/mL. The hexane extract of *V. zizanioides* inhibited 55% of the selected MRSA strains at a relatively low MIC value of 0.012 mg/mL. The hexane extract of both plants demonstrated inhibition of 75% of MRSA strains at a MIC value of 0.064 mg/mL. Ethanol extract of *V. zizanioides* and *E. divaricata,* respectively, inhibited 70% and 45% of MRSA strains at the MIC of 0.096 mg/mL, whereas water extract of *E. divaricata* inhibited 80% of MRSA strains at the same MIC. Both *E. divaricata* and *V. zizanioides* were equally effective against MRSA at a MIC of 0.064 mg/mL. But *V. zizanioides* was more effective since the hexane extract inhibited more than 50% of MRSA strains at significantly a lower MIC value of 0.012 mg/mL. Fractionation, purification, and identification of active compounds will warrant further evaluation of the therapeutic potential of both plant extracts.

## 1. Introduction

Antimicrobial resistance has become a major public health issue in the 21^st^ century which has threatened the prevention and the treatment of a wide variety of infections caused by bacteria, viruses, and fungi. The rate of emergence of antibiotic-resistant human pathogens is greater compared to the discovery of new antibiotic drugs [1]. Among the antibiotic-resistant organisms, methicillin-resistant *Staphylococcus aureus* (MRSA) causes a plethora of diseases involving the skin, soft tissue, bone, and joints [2]. It is one of the major causes of the hospital- and community-acquired infections [2]. The resistance for *β*-lactam antibiotics in MRSA is acquired by the expression of *mecA* gene [3]. Majority of clinical isolates of MRSA are also resistant to clindamycin, ciprofloxacin, and levofloxacin [4].

MRSA infections are prevalent in Sri Lanka mainly due to prophylactic and empiric use of antibiotics [5]. It is highly prevalent not only in hospitals but also in community samples in Sri Lanka [6]. Vancomycin is still the most commonly used antibiotic for multidrug-resistant *S. aureus*, but recent studies report the occurrence of *S. aureus* strains that are resistant to vancomycin in many countries including Sri Lanka [7]. Therefore, an urge of the discovery of new drugs to combat drug-resistant microorganisms including *S. aureus* is essential.

**Figure 1:**
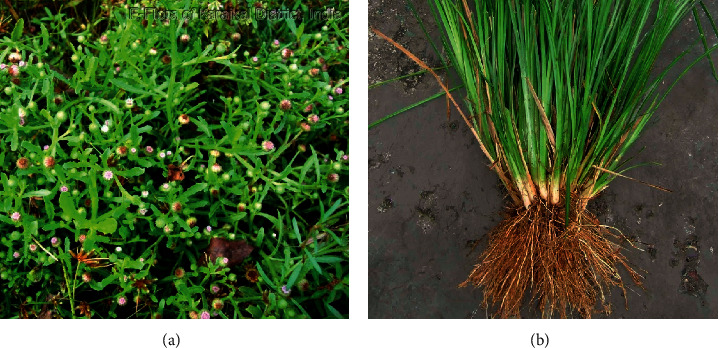
(a) Botanical name: *Epaltes divaricata*, local name: “Heen mudamahana,” family: Compositae; (b) botanical name: *Vetiveria zizanioides*, local name: “Sevendara,” family: Gramineae.

Medicinal plants are a key source of alternative medicine for fighting against diseases since ancient times. They are rich sources of valuable secondary metabolites which are capable of inhibiting microorganisms [8]. These natural products are also promising sources for the synthesis of novel antibacterial compounds. The whole plant of *Epaltes divaricata* and roots of *Vetiveria zizanioides* (Figure 1) are widely used in traditional medicine in Sri Lanka [9]. *Vetiveria zizanioides* (“Sevendara” in Sinhala) is commonly found in the warmer parts of the island. It is being used in Ayurveda for the treatment of typhoid fever, haemoptysis, phthisis, anaemia, skin and blood diseases, urinary disorders, piles, oedema, anorexia, chronic dyspepsia, flatulence, and acute and chronic congestion of the liver and jaundice [10]. *Epaltes divaricata* (“Heen-mudamahana” in Sinhala) is being used to treat skin diseases, oedema, pains, headaches, epilepsy, jaundice, haemorrhoids, hernia, and dysuria [11].

One of our previous studies reported the antibacterial activity of *E. divaricata* and *V. zizanioides* against standard organisms such as *Staphylococcus aureus* (ATCC 25923), *Escherichia coli* (ATCC 25922), *Pseudomonas aeruginosa* (ATCC 27853), and *Klebsiella pneumoniae* (ATCC 700603) [12]. However, *in vitro* studies on these plants for multidrug-resistant organisms are not available. Therefore, this study explores the antibacterial activity against MRSA strains which were isolated from pus samples obtained for culture and sensitivity testing from patients having skin and soft tissue infections.

## 2. Methods

### 2.1. Plant Collection and Authentication

Whole plants of *Epaltes divaricata* and roots of *Vetiveria zizanioides* (Figure 1) were collected from the Southern Province in Sri Lanka. Authentication of the plants was confirmed at the National Herbarium, Botanical Gardens, Peradeniya, Sri Lanka. The plant material was thoroughly washed and dried in a hot air oven at 40°C for 3-4 days. Dried plant material was ground to a coarse powder and stored in sterile airtight containers at 4°C.

### 2.2. Preparation of Plant Extracts

#### 2.2.1. Ethanol and Hexane Extracts

20 g of coarse powder of each plant material was mixed with 200 ml of the solvent. The contents were placed in a mechanical shaker for 72 hr at 25°C. Then, the extracts were filtered, and the solvent was evaporated using a rotary evaporator. The resulting crude extracts were dissolved in a minimum amount of 10% dimethyl sulfoxide (DMSO) separately, and the final concentration of each extract was adjusted to 300 mg/ml. Each extract was stored at 4°C in sterile airtight containers for further studies.

#### 2.2.2. Water Extract

2.62 g of dried plant material was refluxed in 60 ml of distilled water for 3 hrs to obtain the water extract. The resulting crude extract was dissolved in a minimum amount of 10% DMSO, and the final concentration of the extract was adjusted to 300 mg/ml. The extract was stored at 4°C in sterile airtight containers for further studies.

### 2.3. Test Organisms

MRSA strains (*n* = 20) were used for the study along with CLSI (Clinical and Laboratory Standards Institute) standard strain of methicillin-sensitive *S. aureus* (MSSA-ATCC 25923) as the reference organism. The MRSA strains used in this study were isolated from pus samples obtained for culture and sensitivity testing from patients having skin and soft tissue infections among patients admitted to Teaching Hospital, Karapitiya, Sri Lanka. Organisms were subcultured for 24 hours on blood agar and MacConkey agar plates and confirmed by gram stain, catalase test, slide and tube coagulase tests, and the zone diameter of cefoxitin (30 *μ*g) on Mueller–Hinton agar at the Department of Microbiology, Faculty of Medicine, University of Ruhuna, Sri Lanka.

### 2.4. Bacterial Susceptibility Testing

Bacterial susceptibility was tested using the disc diffusion assay. Crude extract, 10-fold, and 100-fold dilutions of each plant extract were prepared in 10% DMSO. Bacteria cell suspensions were adjusted to 0.5 McFarland turbidity standards to prepare 1 × 10^8^ CFU/ml inoculum. Each standardized inoculum was introduced and evenly distributed on the surface of sterile Mueller–Hinton agar plates. Previously prepared sterile filter paper discs (Whatman No. 1, diameter = 6 mm) were soaked in 10 *μL* of each plant extract. They were placed on the seeded Mueller–Hinton agar plates. Vancomycin (30 *μ*g/disc) disk was used as the positive control, and 10% DMSO-soaked filter paper disk was used as the negative control. The same procedure was used for all the MRSA strains used. The plates were incubated aerobically at 35 ± 2°C for 18–24 hr. After incubation, diameters of the zones of inhibition were measured using a Vernier caliper. Each test was carried out in triplicate, and the average values of the diameters were considered. Activity index (AI) and relative percentage inhibition (RPI) for each extract were calculated using the following formula [13]:

(1) $$\begin{array}{c} \text{RPI}=\frac{100\left(X-Y\right)}{\left(Z-Y\right)},\\ \text{AI}=\frac{\text{inhibition zone diameter of the sample}}{\text{inhibition zone of the standard}}\text{,} \end{array}$$

where *X* = total area of inhibition of the test extract, *Y* = total area of inhibition of the solvent, and *Z* = total area of inhibition of the standard drug.

The total area of the inhibition was calculated by using area = *πr*^2^; where *r* = radius of zone of inhibition.

### 2.5. Determination of Minimum Inhibitory Concentration (MIC)

Broth microdilution method was used to determine the MIC. Serial 5-fold dilutions of the plant extracts were prepared in the 10% DMSO, yielding serial dilutions of the crude extract. Bacterial inoculum was prepared in Mueller–Hinton broth, and the turbidity was adjusted to approximately 0.5 McFarland turbidity standards. 96-well microtitre plates were used for the MIC assay, and 150 *μ*L of plant extract was added to each well of the microplate. 50 *μ*L of bacterial suspension was added to each well except the negative controls. Vancomycin (MIC ≤ 2 *μ*g/ml) was used as positive control. 10% DMSO and plant extracts without bacterial suspension were used as the negative controls. Microtiter plates were incubated at 35 ± 2°C for 24 hr. Antimicrobial activity was assessed by the measurement of absorbance at 630 nm using a microplate reader. The lowest concentration (highest dilution) of the extract that does not produce bacterial growth depending on the absorbance was regarded as MIC. The assay was done in triplicate for each extract separately, and average absorbance values were used to determine the MIC.

### 2.6. Ethical Approval

Ethical approval was obtained from the Ethical Review Committee, Faculty of Medicine, University of Ruhuna, Sri Lanka.

### 2.7. Statistical Analysis

Mean standard error of means (SEM) was calculated to express data. The data were analysed using one-way ANOVA to compare the mean between groups. Mean differences at each point were considered significant at *p* < 0.05.

## 3. Results

**Figure 2:**
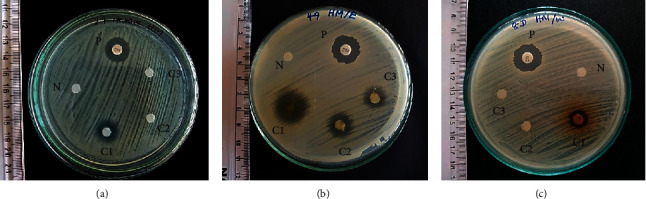
Inhibitory zones of crude extracts of *E. divaricata*: (a) hexane extract; (b) ethanol extract; (c) water extract. P: positive control; N: negative control; C1: crude extract; C2: 10-fold dilution of crude extract; C3: 100-fold dilution of crude extract.

**Figure 3:**
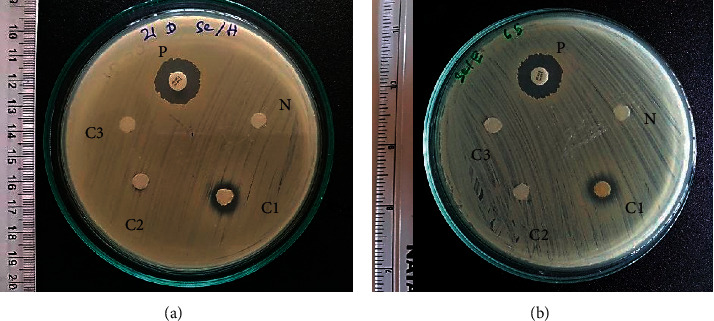
Inhibitory zones of crude extracts of *V. zizanioides*: (a) hexane extract; (b) ethanol extract. P: positive control; N: negative control; C1: crude extract; C2: 10-fold dilution of crude extract; C3: 100-fold dilution of crude extract.

**Figure 4:**
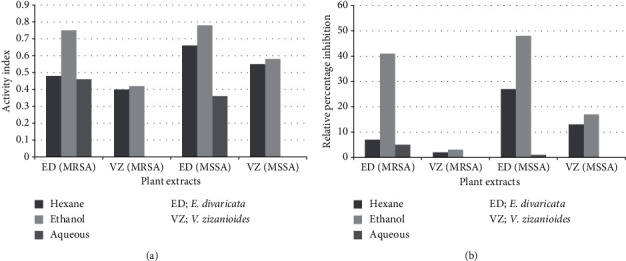
Activity index (a) and relative percentage inhibition (b) of crude extracts of *E. divaricata* and *V. zizanioides* for MRSA and MSSA.

Disc diffusion assay was performed to detect the presence of inhibitory zones of *E. divaricata* and *V. zizanioides* against MRSA. Inhibitory zones were observed from crude extracts (hexane, ethanol, and water) of *E. divaricata* against MRSA (Figure 2 and Table 1). Except for water extract, both hexane and ethanol extracts of *V. zizanioides* showed inhibitory zones (Figure 3). Relatively larger inhibitory zones ranging from 13.1 to 18.7 mm were observed from the undiluted crude ethanol extract of *E. divaricata*, while diameters of inhibitory zones of water and hexane extracts were lower ranging from 6.6 mm to 15.6 mm and 6.7 mm to 13.1 mm, respectively. Similarly, in *V. zizanioides*, larger inhibitory zones were observed from undiluted ethanol extract ranging from 6.4 mm to 13.3 mm followed by hexane extract which showed a diameter range of 6.5–11.4 mm. Highest AI and RPI values were observed from crude ethanol extracts of both plants (Figure 4). Among diluted extracts of *E. divaricata*, inhibitory zones were observed from 10-fold to 100-fold diluted ethanol extracts. In *V. zizanioides*, only the 10-fold diluted ethanol extract showed inhibitory zones. Mean inhibitory zone of vancomycin (30 *μ*g/disc) for MRSA and MSSA was 20.6 mm and 20.8 mm, respectively. Compared to the MRSA strains, MSSA (ATCC 25923) also showed similar susceptibility pattern for the corresponding plant extracts (Table 1).

MIC ranges of hexane, ethanol, and water extracts in *E. divaricata* were 0.012–0.32 mg/mL, 0.019–2.4 mg/mL, and 0.019–0.48 mg/mL, respectively. Respective MIC ranges of hexane and ethanol extracts of *V. zizanioides* were 0.003–0.032 mg/mL and 0.019–2.4 mg/mL. The hexane extract of *V. zizanioides* inhibited 75% of the selected MRSA strains at relatively low MIC values ranging from 0.003 to 0.064 mg/mL. The hexane extract of *E. divaricata* inhibited 75% of MRSA strains at MIC values ranging from 0.012 mg/mL to 0.064 mg/mL. Ethanol extract of *V. zizanioides* inhibited 70% of MRSA strains at the MIC values ranging from 0.019 mg/mL to 0.096 mg/mL, whereas ethanol and water extracts of *E. divaricata,* respectively, inhibited 45% and 80% of MRSA strains at the same MIC range. Relatively high MIC values of ethanol extracts of *E. divaricata* and *V. zizanioides* ranging from 0.48 to 2.4 mg/mL were needed to inhibit 55% and 30% of selected MRSA strains, respectively. Similarly, 0.48 mg/mL concentration of the water extract of *E. divaricata* was needed to inhibit 20% of selected MRSA strains (Tables 2 and 3).

MRSA which were not effectively inhibited by extracts of *E. divaricata* was effectively inhibited by extracts of *V. zizanioides*, and vice versa. MRSA nos. 2 and 3 were inhibited at MIC of 2.4 mg/mL of the ethanol extract of *E. divaricata*, but they were inhibited at 0.012 mg/mL by the hexane extract of *V. zizanioides*. MRSA nos. 9 and 10 that were inhibited at MIC of 0.32 mg/mL of hexane extract of *V. zizanioides* were inhibited at 0.012 mg/mL by the hexane extract of *E. divaricata*. Furthermore, MIC values obtained for the different extracts of the same plant for individual MRSA also varied. MRSA no. 6 which was inhibited at MIC of 0.48 mg/mL of ethanol extract was also inhibited at MIC of 0.012 mg/mL of hexane extract of *V. zizanioides*. MRSA no. 9 was inhibited at MIC of 2.4 mg/mL of ethanol extract, but it was also inhibited at MIC of 0.012 mg/mL of hexane extract of *E. divaricata*. The reference strain of *S. aureus* (ATCC 25923) was more sensitive for the hexane extract of *V. zizanioides* which required MIC of 0.003 mg/mL. Relatively high MIC values ranging from 0.48 to 2.4 mg/mL of ethanol and hexane extracts were needed to inhibit *S. aureus* (ATCC 25923) (Table 2). There were no statistically significant differences in MIC between hexane, ethanol, and aqueous extracts of *E. divaricata* and *V. zizanioides* (*p* > 0.05).

## 4. Discussion

The results revealed that the crude extracts of *E. divaricata* and *V. zizanioides* were effective against the MRSA strains as observed by the zones of inhibition. Disc diffusion assay was performed as a preliminary screening to confirm the inhibitory effects of the plant extracts against MRSA. Highest AI and RPI values were obtained from crude ethanol extracts of the plants. Since undiluted crude extracts were more effective against MRSA, it is assumed that the antibacterial effect may be due to the presence of possible active ingredients in high concentrations. However, the strength of an inhibitory effect cannot be compared by the results of disc diffusion assay due to the varying concentrations of the crude extracts. Therefore, MIC was determined as the standard method to compare the antibacterial activity of the plant extracts.

The MIC values obtained for the extracts against the MRSA varied from one plant extract to another. One reason would be the extraction of different constituents in particular solvents depending on their chemical and physical properties especially the polarity. Furthermore, due to genetic variability, different MRSA could have acted differently with chemicals in each extract. Organisms which were not effectively inhibited by *E. divaricata* were effectively inhibited by extract of *V. zizanioides*, and vice versa, suggesting the effectiveness of both plants towards different strains of MRSA. However, *V. zizanioides* is more effective since hexane extracts inhibited majority of MRSA strains at relatively lower MIC such as 0.003 mg/mL. Low polar active compounds present in the hexane extract may be responsible for this antimicrobial activity. As observed in our previous studies, compared to the MRSA strains, *S. aureus* ATCC standard (ATCC 25923) also showed similar susceptibility pattern for both plants [12]. This could be due to the genetic similarities between MSSA (ATCC 25923) and MRSA.

Studies conducted in Sri Lanka revealed that certain medicinal plants which are being used in traditional medicine possess anti-MRSA properties [14, 15]. However, local studies on *E. divaricata* and *V. zizanioides*, related to MRSA, were deficient. On the contrary, very few studies were available from other countries on these plants which reported anti-MRSA activity. Glorybai et al. reported the presence of antibacterial activities of *E. divaricata* in 2015, but they have used a single strain of MRSA, and MIC was not reported [16]. Furthermore, their study revealed the presence of phenolic compounds, 2-butenamide, *N*-(4-fluorophenyl)–methyl *trans*-cinnamyl tiglate silane, trichlorocyclohexyl silane, and its derivatives in *E. divaricata*, which could be responsible for the antibacterial activity. Sivagurunathan and Krishnamoorthy reported the antibacterial properties of *V. zizanioides* for a single strain of MRSA in 2017, but MIC was not reported. High concentrations of phenolic compounds were identified in the ethanol extract, and HPLC results showed the presence of different types of unidentified phytoconstituents [17]. Zuo et al. reported anti-MRSA activity of 19 medicinal plants against 9 MRSA strains (except *E. divaricata* and *V. zizanioides*) with MIC range of 1.25–3.07 mg/ml [18].

There were many reports in the recent past on the antimicrobial activity of medicinal plants against many microorganisms including MRSA. Dahiya and Purkayastha reported antibacterial activity of seven medicinal plants for a single strain of MRSA in 2012 [19]. Extracts of Tulsi, oregano, rosemary, and *Aloe vera* were included in their study, and MIC values were found to be in the range of 1.56–6.25 mg/ml. When compared to other studies conducted by Okwu et al., Zuo et al., and Dahiya and Purkayastha [18–20], extracts of *E. divaricata* and *V. zizanioides* in the present study exhibited relatively lower MICs against MRSA.

Trong Le et al. reported antimicrobial activities of essential oils extracted from *Paramignya trimera* and *Limnocitrus littoralis* which were used in the Vietnamese traditional medicine [21]. *P. trimera* strongly inhibited *S. aureus* ATCC 43300 and the *S. aureus* clinical strain with MIC and MLC (minimum lethal concentration) values of 2% (v/v), but *L. littoralis* did not inhibit *S. aureus*. Oil extracted from *P. trimera* consisted of *β*-caryophyllene, *β*-caryophyllene oxide, 7-epi-*α*-eudesmol, and *γ*-muurolene as major components. This suggested that the presence of *β*-caryophyllene and *β*-caryophyllene oxide may be responsible for the antibacterial activity of *P. trimera* against *S. aureus.* Another Vietnamese study on essential oil extracted from the leaves of *Leoheo domatiophorus* inhibited *S. aureus* ATCC 43300 and the *S. aureus* clinical strain [22]. Both *S. aureus* strains were inhibited at MIC of 0.25% (v/v) and MLC of 0.5% (v/v). GC/MS (gas chromatography/mass spectrometry) analysis of the essential oil of *L. domatiophorus* indicated the presence of sesquiterpene hydrocarbons, oxygenated sesquiterpenes as the main classes of compounds. Viridiflorene, (−)-*δ*-cadinene, and *γ*-muurolene were present at higher concentrations, while *α*-muurolene, *γ*-cadinene, (+)-aromadendrene, *α*-cadinol, and globulol were present at lower concentrations.

Many other studies published recently also reported the presence of compounds of natural origin that could alleviate the symptoms associated with skin infections. A study conducted on water extract of *Borojoa patinoi* which was used in Colombian traditional medicine did not inhibit *S. aureus* M121 which is resistant to both methicillin and vancomycin [23]. However, the extract inhibited multidrug-resistant (MDR) *Pseudomonas aeruginosa* and the UHPLC (ultrahigh performance liquid chromatography) analysis of the plant extract showed the presence of 26 phenolic compounds including hydroxycinnamic acids, phenolic acids, flavonols, flavan-3-ols, flavonones, flanones, tyrosol ester, and dihydrochalcones. Mazzarello et al. reported that a skin cream containing propolis 20%, tea tree oil 3%, and *Aloe vera* 10% was effective in reducing acne [24]. Pathogenesis of acne is associated with *Propionibacterium acnes*, *S. aureus*, and *S. epidermidis.* Propolis consists of flavonoids, caffeic acid, benzoic acid, and cinnamic acid. Tea tree oil contains terpinen-4-ol, which shows an important role in the antimicrobial activity. Besides, another study on acne reported antiacne efficacy of two essential oils extracted from *Origanum vulgare* and *Myrtus communis* L. [25]. Presence of monoterpene, diterpene, sesquiterpene hydrocarbons, azulene, alcohols, aldehydes, and ketones in essential oils may have attributed to the antibacterial effects against MDR bacteria [26].

One of our previous studies also confirmed the presence of tannins, phenolic compounds, cardiac glycosides, flavonoids, alkaloids, and saponins in both *E. divaricata* and *V. zizanioides* plant extracts [12]. Dos Santos et al. reported that *β*-vetivenene, khusimol, vetiselinenol, isovalencenol, vetivenic acid, *α*-vetivone, and *β*-vetivone are the major constituents of dichloromethane fraction of *Vetiveria zizanioides* [27]. Antimicrobial activity of plant phenolics including flavonoids has also been documented in the past [28]. As evident from the abovementioned studies, active compounds isolated from medicinal plants have a significant potential to alleviate the symptoms associated with skin infections. Composition of active compounds in these plants could vary from one country to another due to different geographical and environmental conditions. Antimicrobial activities could be enhanced if the active compounds of the species under study that were collected from Sri Lanka are purified [29]. Therefore, further investigations which involve fractionation, purification, and identification of active compounds are necessary to develop these two plants as potential therapeutic agents in the future. Previous studies conducted in our laboratory reported the absence of any toxicological effects (biochemical, haematological, and histopathological) in ICR mice when treated with water extracts of *E. divaricata* and *V. zizanioides* [9]. However, toxicological studies on hexane and ethanol extracts have not been conducted yet. To develop potential therapeutics with anti-MRSA properties from these plant extracts, the toxicity of active compounds and their pharmacokinetics should be investigated. *In vivo* studies including clinical trials are necessary to confirm the efficacy of active compounds.

## 5. Conclusion and Recommendation

Here, we present preliminary findings of antibacterial activities of crude extracts of *E. divaricata* and *V. zizanioides* against twenty clinical isolates of MRSA. Both plants are equally effective against MRSA at a MIC of 0.064 mg/mL, but *V. zizanioides* is more effective since the hexane extract inhibited more than 50% of MRSA strains at significantly lower MIC of 0.012 mg/mL. Presence of phenolic compounds, alkaloids, and flavonoids identified in a previous study confirmed the presence of possible active compounds responsible for the antimicrobial effect in both *E. divaricata* and *V. zizanioides*. Since hexane extract was proven as the most active extract, low polar active phytochemicals present in the plant extracts may be responsible for this antimicrobial activity. Fractionation, purification, and identification of active compounds are essential in developing these extracts as potential therapeutics. *In vivo* studies will be performed with purified active components to investigate their pharmacokinetics and toxic effects in the future.

## Figures and Tables

**Table 1:** Diameter values of the disk diffusion assay of *E. divaricata* and *V. zizanioides* against MSSA and MRSA strains.

Extract	Concentration	Inhibition zone diameter (mm)			
		MRSA		MSSA (ATCC 25923)	
		*E. divaricata*	*V. zizanioides*	*E. divaricata*	*V. zizanioides*
Hexane	40 mg/mL (crude)	9.9 (6.7–13.1)^*∗*^, *n* = 20	8.3 (6.5–11.4), *n* = 20	13.7 ± 0.1^#^	11.4 ± 0.2
	4 mg/mL (10x dilution)	—	—	—	—
	0.4 mg/mL (100x dilution)	—	—	—	—

Ethanol	300 mg/mL (crude)	15.4 (13.1–18.7), *n* = 20	8.7 (6.4–13.3), *n* = 8	16.3 ± 0.2	12.1 ± 0.2
	30 mg/mL (10x dilution)	10.1 (7.4–11.7), *n* = 18	6.8 (6.3–9.0), *n* = 8	13.4 ± 0.2	7.3 ± 0.3
	3 mg/mL (100x dilution)	7.1 (6.3–10.1), *n* = 8	—	7.6 ± 0.1	—

Aqueous	150 mg/mL (crude)	9.4 (6.6–15.6), *n* = 20	—	7.4 ± 0.2	—
	15 mg/mL (10x dilution)	—	—	—	—
	1.5 mg/mL (100x dilution)	—	—	—	—

Vancomycin	30 *μ*g/disc	20.6 ± 2.2^†^		20.8 ± 0.1^#^	

*n* denotes the number of isolates that showed a measurable diameter; ^*∗*^data were expressed as mean (min-max); ^#^data were expressed as mean ± SEM; ^†^mean ± standard deviation.

**Table 2:** MIC values of *E. divaricata* and *V. zizanioides* against MRSA strains.

MRSA strain	Minimum inhibitory concentration (mg/mL)				
	Hexane extract		Ethanol extract		Water extract
	*E. divaricata*	*V. zizanioides*	*E. divaricata*	*V. zizanioides*	*E. divaricata*
1	0.064	0.064	0.09	0.48	0.09
2	0.064	0.012	2.40	0.09	0.09
3	0.32	0.012	2.40	0.09	0.48
4	0.32	0.32	0.09	0.09	0.48
5	0.32	0.012	0.48	0.09	0.09
6	0.32	0.012	0.48	048	0.09
7	0.064	0.003	0.09	0.09	0.09
8	0.32	0.064	0.48	2.40	0.48
9	0.012	0.32	0.48	2.40	0.48
10	0.012	0.32	2.40	0.48	0.09
11	0.064	0.012	0.48	0.09	0.09
12	0.012	0.012	0.019	0.019	0.09
13	0.064	0.003	0.09	0.09	0.09
14	0.064	0.064	0.48	0.09	0.09
15	0.064	0.012	0.09	0.019	0.09
16	0.064	0.32	0.09	0.09	0.019
17	0.064	0.012	0.09	0.019	0.09
18	0.012	0.32	0.48	0.09	0.09
19	0.012	0.064	0.48	0.09	0.019
20	0.064	0.012	0.09	0.48	0.09

Mean (SEM) *n* = 20	0.064^*∗*^ (0.012–0.32)	0.098^*∗*^ (0.003–0.32)	0.480^*∗*^ (0.019–2.4)	0.388^*∗*^ (0.019–2.4)	0.399^*∗*^ (0.019–0.48)
MSSA^#^	1.6	0.003	0.48	2.4	1.2

^*∗*^Data were expressed as mean (min-max); ^#^methicillin-sensitive *S. aureus* (ATCC 25923).

**Table 3:** Percentage of MRSA strains inhibited at different MIC values of *E. divaricata* and *V. zizanioides*.

MIC (mg/mL)	Percentage (%) of MRSA inhibited				
	Hexane extract		Ethanol extract		Water extract
	*E. divaricata*	*V. zizanioides*	*E. divaricata*	*V. zizanioides*	*E. divaricata*
0.003	—	10% (*n* = 2)	—	—	—
0.012	25% (*n* = 5)	45% (*n* = 9)	—	—	—
0.019	—	—	5% (*n* = 1)	15% (*n* = 3)	10% (*n* = 2)
0.064	50% (*n* = 10)	20% (*n* = 4)	—	—	—
0.096	—	—	40% (*n* = 8)	55% (*n* = 11)	70% (*n* = 14)
0.320	25% (*n* = 5)	25% (*n* = 5)	—	—	—
0.480	—	—	40% (*n* = 8)	20% (*n* = 4)	20% (*n* = 4)
2.400	—	—	15% (*n* = 3)	10% (*n* = 2)	—

Total	100% (*n* = 20)	100% (*n* = 20)	100% (*n* = 20)	100% (*n* = 20)	100% (*n* = 20)

*n* = number of MRSA strains.

## Data Availability

The data used to support the findings of the present study are available from the corresponding author upon request.
